# PTPRC promoted CD8+ T cell mediated tumor immunity and drug sensitivity in breast cancer: based on pan-cancer analysis and artificial intelligence modeling of immunogenic cell death-based drug sensitivity stratification

**DOI:** 10.3389/fimmu.2023.1145481

**Published:** 2023-06-14

**Authors:** Pengping Li, Wei Wang, Shaowen Wang, Guodong Cao, Tonghe Pan, Yuqing Huang, Hong Wan, Weijun Zhang, Yate Huang, Haigang Jin, Zhenyu Wang

**Affiliations:** ^1^ Department of Thyroid & Breast Surgery, The First People’s Hospital of Xiaoshan District, Xiaoshan Affiliated Hospital of Wenzhou Medical University, Hangzhou, Zhejiang, China; ^2^ Department of Oncology, The Second Affiliated Hospital of Bengbu Medical College, Anhui, China; ^3^ Neuromedicine Center, The University of Hong Kong-Shenzhen Hospital, Shenzhen, Guangdong, China; ^4^ The Department of General Surgery, The First Affiliated Hospital of Anhui Medical University, Hefei, Anhui, China; ^5^ The Department of Ningbo Eye Hospital, Affiliated to Wenzhou Medical University, Ningbo, Zhejiang, China; ^6^ Anhui Public Health Clinical Center, The Fourth Affiliated Hospital of Anhui Medical University, Hefei, China; ^7^ School of Ophthalmology and Optometry, Wenzhou Medical University, Wenzhou, Zhejiang, China

**Keywords:** immunogenic cell death (ICD), drug sensitivity, breast cancer, PTPRC, CD8+ T cell

## Abstract

**Background:**

Immunogenic cell death (ICD) is a result of immune cell infiltration (ICI)-mediated cell death, which is also a novel acknowledgment to regulate cellular stressor-mediated cell death, including drug therapy and radiotherapy.

**Methods:**

In this study, TCGA and GEO data cohorts were put into artificial intelligence (AI) to identify ICD subtypes, and in vitro experiments were performed.

**Results:**

Gene expression, prognosis, tumor immunity, and drug sensitivity showed significance among ICD subgroups, Besides, a 14-gene-based AI model was able to represent the genome-based drug sensitivity prediction, which was further verified in clinical trials. Network analysis revealed that PTPRC was the pivotal gene in regulating drug sensitivity by regulating CD8+ T cell infiltration. Through in vitro experiments, intracellular down-regulation of PTPRC enhanced paclitaxel tolerance in triple breast cancer (TNBC) cell lines. Meanwhile, the expression level of PTPRC was positively correlated with CD8+ T cell infiltration. Furthermore, the down-regulation of PTPRC increased the level of TNBC-derived PD-L1 and IL2.

**Discussion:**

ICD-based subtype clustering of pan-cancer was helpful to evaluate chemotherapy sensitivity and immune cell infiltration, and PTPRC was a potential target to against drug resistance of breast cancer.

## 1 Introduction

During the multi-stage development process of cancer, immune surveillance, an immune process that recognizes and kills numerous precancerous or cancerous cells, is generally considered to regulate the normal cell differentiation, cancer cell proliferation, and cell death modalities (1). With the development of cancer, cancer cells gradually develop a variety of different strategies (such as acquiring defects in antigen presentation mechanism) to escape the immune surveillance and create an environment suitable for their proliferation, which is also termed as the tumor microenvironment (TME) (2). The complex TME ecosystem is composed of various immune cells and cancer cells. The infiltration of immune cells plays a key role in tumor development and therapeutic response. Some types of the immune cells in the TME can lead to tumor immune escape by inhibiting the anti-tumor immune response (3, 4).

Immunogenic cell death (ICD) is a type of regulated cell death with different functions, which refers to the secretion and exposure of damage-related molecular patterns (DAMPs) from dying tumor cells in the tumor microenvironment (TME) to the immunosuppressed TME to re-establish the immune surveillance. And it can engage both the innate and adaptive immunities to activate tumor-specific immune responses (5–9). DAMPs include the cell surface exposure to calreticulin (CRT), heat-shock proteins (HSP70 and HSP90), extracellular ATP, high mobility group box-1 (HMGB1) and type I interferons (IFNs), etc. (2, 10). During ICD, the released DAMPs can bind to specific pattern recognition receptors (PRRs) expressed by dendritic cells (DCs) and initiate a cellular cascade, eventually leading to the activation of both innate and adaptive immune responses (5, 11, 12).

Therefore, in the context of anti-cancer treatment, triggering ICD is clinically significant based on its inherent role to enhance the therapeutic effect by recruiting anti-tumor immune cells (7). Clinically, drug-induced ICD is reported to positively correlate with therapeutic response and is associated with immune enhancement of TME (7, 13–16). However, most of the current studies focus on the possibility and mechanism of using ICD to treat pan-cancer, and the use of ICD in TME to predict drug sensitivity and prognosis in pan-cancer treatment has not been well studied (7).

In this study, we aimed to construct a drug-sensitivity and prognosis model for pan-cancer and analyze its relationship with TME and ICD. Furthermore, we identified PTPRC as a pivotal ICG-associated tumor driver gene (ICD-TDG) in regulating the CD8+ T cell infiltration-mediated anti-tumor effect.

## 2 Materials and methods

**Figure 1:**
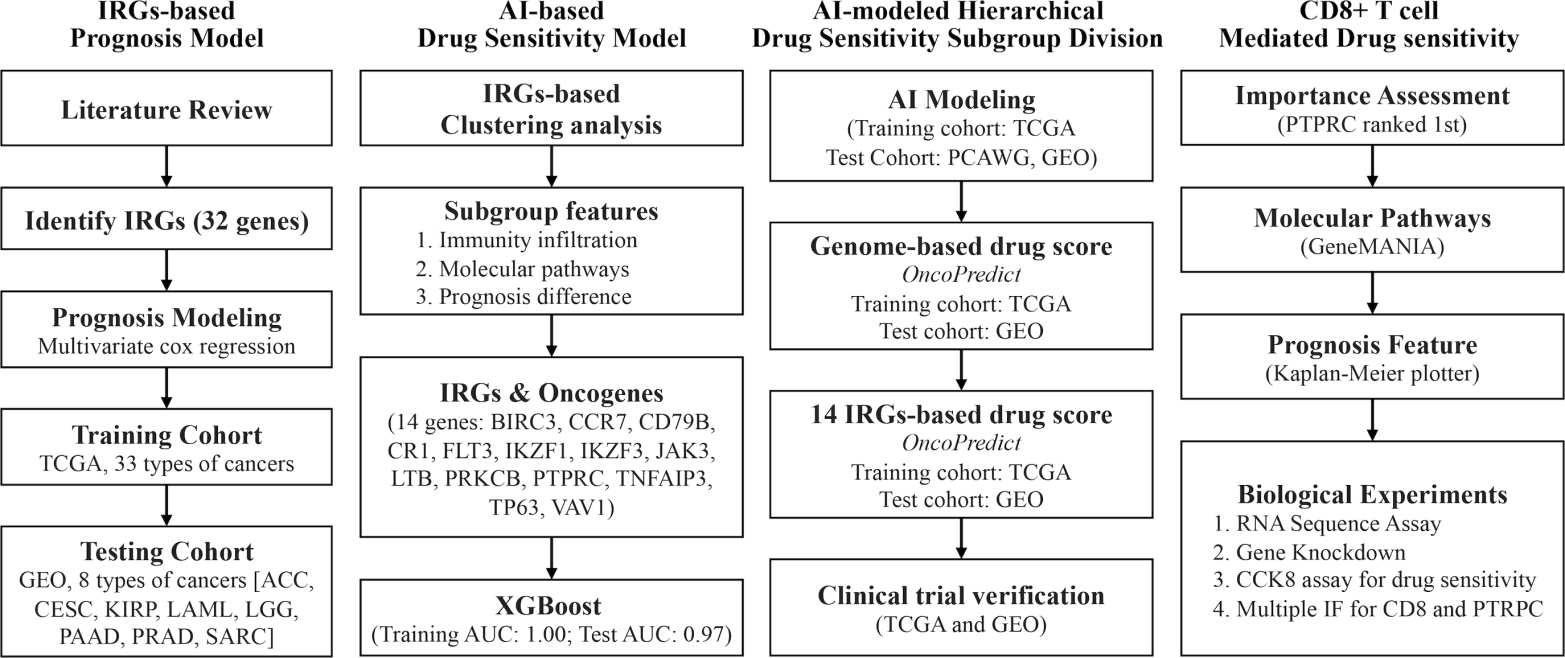
Technology roadmap of study. 32 ICD-associated genes were collected from previous studies and used to construct a prognosis model in the TCGA cohort and the GEO cohort; 14 IRGs-related TDGs were used to construct AI models based on IRGs-based clustering; 14 IRGs-TDGs-based and genome-based drug sensitivity prediction; The role of PTPRC in regulating CD8+ T cells infiltration-mediated chemotherapy sensitized.

This study explored the significance of immunogenic cell death (ICD)-related genes (IRGs) in identifying breast cancer subgroups with different drug sensitivity in chemotherapy and anti-endocrine therapy. The whole research process of this study was shown in **Figure 1**. The whole data analysis process was performed by two researchers, respectively.

### 2.1 Data collection

Gene expression profiles and clinical characteristics of pan-cancer were collected from UCSC Xena (http://xena.ucsc.edu), and 9593 samples were finally included. Breast cancer gene expression profiles were collected from The Cancer Genome Atlas (TCGA, https://portal.gdc.cancer.gov) and Gene Expression Omnibus (GEO: https://www.ncbi.nlm.nih.gov/geo/), amongst which 1089 samples with follow-up data were collected from TCGA, and 409 samples with follow-up data were collected from GSE58812, GSE7390, GSE42918. Besides, gene expression profiles of 1084 samples without subtypes division were collected from GSE51561, GSE20685, GSE20711, GSE25066, GSE29431, and GSE61304, and gene expression profiles of 517 triple negative breast cancer (TNBC) samples were collected from GSE18864, GSE58812, GSE76124, GSE83937, GSE97500. 1548 clinical trial sample data involved in drug sensitivity were collected from GSE25066, GSE25055, GSE25065, GSE32646, GSE50948, GSE66305, GSE22093, GSE25066, GSE20194, GSE20271, GSE42822, and GSE23988. Verified cohorts of ACC were from GSE10927, GSE19750, GSE33371, GSE76019, and GSE76021, a verified cohort of CESC was from GSE44001, a verified cohort of KIRP was from International Cancer Genome Consortium (ICGC, http://dcc.icgc.org), a verified cohort of LAML was from GSE37642, a verified cohort of LGG were from mRNAseq_693/325 (Chinese Glioma Genome Atlas, CGGA, http://www.cgga.org.cn), verified cohorts of PAAD were from GSE21501, GSE28735, GSE57495, and GSE62452, a verified cohort of PRAD was from GSE116918, and verified cohort of SARC was from GSE21050. Data cleaning and data combination were performed in online tools (Sangerbox 3.0 (17), http://vip.sangerbox.com/home.html). 32 ICD-related genes were obtained from previous research (gene list: ATG5, BAX, CALR, CASP1, CASP8, CD4, CD8A, CD8B, CXCR3, EIF2AK3, ENTPD1, FOXP3, HSP90AA1, IFNB1, IFNG, IFNGR1, IL10, IL17A, IL17RA, IL1B, IL1R1, IL6, LY96, MYD88, NLRP3, NT5E, P2RX7, PDIA3, PIK3CA, PRF1, TLR4, TNF) (18). 586 tumor driver genes were collected from Lopez-Bigas N’s research (19). The relationship between gene expression and prognosis in BRCA was obtained from online tools (Kaplan Meier-plotteR: http://kmplot.com/analysis/index.php?p=background). The PTPRC-related genes were collected from The University of Alabama at Birmingham Cancer data analysis Portal (UALCAN: http://ualcan.path.uab.edu/index.html) and GeneMANIA (http://genemania.org/search/homo-sapiens).

### 2.2 Bioinformatic analysis

#### 2.2.1 IRGs-based subgroups identification

*ConsensusClusterPlus* package in R4.2.0 was used to perform consensus clustering analysis, based on the IRGs (*parameter: maxK=5, reps=50*). Consensus cumulative density function (CDF) and delta area showed 4 subgroups division was the best outcome (**Figure S1**). R codes were showed in the “S1” file.

#### 2.2.2 Principal co-ordinates analysis

PCoA was performed in OEBIOTECH online tools (https://cloud.oebiotech.com/task/), which platform supplied biotechnical support for lots of great research (20–22).

#### 2.2.3 Tumor immune index calculation

Infiltration immune cell fractions were predicted in CIBERSORT in R4.2.0, and the immune score was predicted by the *estimate* package in R4.2.0. R codes were showed in the “S2” file.

#### 2.2.4 ICD-TDGs-based artificial intelligence model of ICD stratification

Firstly, the *limma* package was performed to identify different expression genes between different ICD subgroups (p<0.05 and |logFc|>1.5). Then, the *upset* package was performed to identify genes that were differentially expressed between any two ICD subgroups. Following, put those above-identified genes into a univariate cox regression analysis to identify the prognosis-related one. Finally, making intersection between those genes and 568 cancer driver genes, by which 14 genes (tumor driver genes involved in ICD, ICD-TDGs) were identified (BIRC3, CCR7, CD79B, CR1, FLT3, IKZF1, IKZF3, JAK3, LTB, PRKCB, PTPRC, TNFAIP3, TP63, VAV1). AI modeling for ICD stages was developed by six AI functions, including extreme gradient boosting (XGboost, *xgboost* package in R4.2.0), support vector machine (SVM, *e1071* packages in R4.2.0), multi-logistic (*nnet* packages in R4.2.0), random forest (RF, *randomForest* package in R4.2.0), deep learning (DL, *h2o* package in R4.2.0) and K-Nearest Neighbor (KNN, *kknn* package in R4.2.0). All R codes were showed in the “S3” file. During the model construction, randomly select 75% as the training cohort, and randomly select 25% as the testing cohort. Gene expression value was standardized to range “0~1” with *preProcess* function (*caret* and *tidyverse* packages).

#### 2.2.5 Drug sensitivity prediction

Drug sensitivity prediction was performed with genome or ICD-TDGs by the *oncoPredict* package in R4.2.0, which was used in the previous study (23, 24).

### 2.3 Biological experiments

#### 2.3.1 Clinical sample collecting

5 breast cancer and 3 adjacent fresh frozen samples were collected from 2022-06.01 to 2022-12.31, and 17 breast cancer tissue and 3 adjacent tissue paraffin sections were collected from 2022-06.01 to 2022-12.31. All of the above experiments were approved by the Medical Ethics Committee of The First People’s Hospital of Xiaoshan District, Xiaoshan Affiliated Hospital of Wenzhou Medical University. All patients with breast cancer were confirmed by at least two pathologists.

#### 2.3.2 RNA sequence assay

Breast cancer tissues and adjacent tissues were collected from the surgical operation, and they were washed with 0.9% normal saline within 15 mins, which was followed by liquid nitrogen quick freezing for 20 mins, finally, tissues were stored at -80°C. RNA sequence assay was performed by GENE DENOVO (Guangzhou, China).

#### 2.3.3 Multiple immune fluorescence staining

Experiments procedure of paraffin embedding, tissue section, and immunohistochemistry for PTPRC and CD8 expression level were performed as previously described (PMID: 23200678 and 20571492). What’s more, the work concentration of antibodies against PTPRC (Proteintech, Wuhan, China) and CD8 (Abcam, shanghai) was 1:150. The protein expression level was assessed by Mean of Integrated Option Density (IOD) with Image-Pro Plus. Briefly, area of Interesting (AOI) and detect IOD to gain Mean of IOD (IOD/AOI, MI).

#### 2.3.4 Reagents

Paclitaxel was purchased from CSNpharm (CSN19486, USA), and dissolved in DMSO. Antibodies against GAPDH (5174, CST), CD8 (GB15068, Servicebio), PTPRC (GB113885, Servicebio), PD-L1 (66248-1-Ig, Proteintech), IL2 (60306-1-Ig, Proteintech), and IL6 (21865-1-AP, Proteintech) were used for western blot.

#### 2.3.5 Cell culture

Triple-negative breast cancer cell lines (MBA-MD-231, MBA-MD-453) were gained from the cell bank of the Chinese Academy of Science in 2022 with STR matching analysis. The culture media of MBA-MD-231 was DMEM within 10% fetal calf serum and 100 units/mL penicillin and streptomycin. The culture media of MBA-MD-231 was L-15 within 10% fetal calf serum and 100 units/mL penicillin and streptomycin.

#### 2.3.6 Cell cytotoxicity assays

The cell proliferation was quantified by standard curve (0.1, 0.2, 0.4, 0.8, 1.0, 1.5, 2.0, 3.0×104 cells were detected optical density (OD) *via* CCK-8 after 24h transplanted into 96-wells plates, and then fit linear standard curve between log [cell quantity] and OD), cell cytotoxicity assays were performed *via* CCK8 assay, and the detailed protocol described in our previous study (PMID29331423 and PMID29800682).

#### 2.3.7 Quantitative real-time PCR

Trizol RNA isolation system (Invitrogen, USA) was used for total RNA extraction. The cDNA templates were synthesized through PrimeScript RT Reagent Kit (TaKaRa, China), and qRT-PCR was performed with a 7500 Fast™ System (Applied Biosystems, USA) using the Sensi Mix SYBR Kit (Bio-Rad, USA). The mRNA level was calculated *via* using (=2-ΔΔCt), and normalized to GAPDH. All of the sequences of primer were designed by Primer 5 soft:

*PTPRC-QF: CTTCAGTGGTCCCATTGTGGTG*
*PTPRC-QR: CCACTTTGTTCTCGGCTTCCAG*
*GAPDH-QF: GTCTCCTCTGACTTCAACAGCG*
*GAPDH-QR: ACCACCCTGTTGCTGTAGCCAA*

#### 2.3.8 Small interfering RNA experiments

5 × 10^5^ breast cancer cells were transplanted into 6 wells plates for 24h, and then cells were transfected with three different sequences PTPRC siRNA (GenePharma, Shanghai, China) for 48h, 72h, and 96h with Lipofectamine 3000 reagent (Invitrogen, USA) and Opti-MEM (Life Technologies, USA), according to the manufacturer’s instructions for gaining the best transfection efficiency. Three siRNA sequences for PTPRC were listed in the following:

*siRNA-1: 5’-3’ GACAGGGCAAAGCCCAACAtt; 3’-5’ UGUUGGGCUUUGCCCUGUCtt*
*siRNA-2: 5’-3’UUGGCAUUUGGUUUGCCUtt 3’-5’ AGGCAAAGCCAAAUGCCAAtt*
*siRNA-3:5’-3’ CUUAGGGACACGGCUGACUtt 3’-5’ AGUCAGCCGUGUCCCUAAGtt*
*Recombinant plasmid transfection assay*

Primers of PTPRC is inserted into plasmid pcDNA 3.0 (Addgene), were designed with Primer 5 soft. Briefly, cDNA templates were synthesized through PrimeScript RT Reagent Kit (TaKaRa, China); CDS of genes were amplified with PrimeSTAR^®^ GXL DNA Polymerase (TaKaRa, China); thirdly, products were purified through SanPrep Column DNA Gel Extraction Kit (Sangon Biotech, China); fourthly, the purified products and plasmid were treated with restriction endonuclease (Xho1, EcoR5, and Xba1 were purchased from NEB, USA) respectively; fifthly, recombination of plasmids was performed through homologous recombination with Hieff CloneTM Plus One Step Cloning Kit (Yeasen Biotech, China). 5 × 105 cells were transplanted into 6 wells plates for 24h, and then cells were transfected with RP for 48h, 72h and 96h with Hieff TransTM Liposomal Transfection Reagent (Yeasen Biotech, China) for the best transfection efficiency, according to the manufacturer’s instructions.

#### 2.3.9 Western blot

Total protein extraction: Cells were harvested by cytology brush, and lysed with RIPA lysis buffer (Sigma, USA) supplemented with phosphorylase and protease inhibitor mixture (Thermo, USA), quantified by the BCA assay.

Cytoplasmic and nucleus protein extraction: Cells were harvested by Tyrisin (Invitrogen), then cytoplasmic and nucleus protein was extracted by Cytoplasmic and Nucleus Protein Extraction Kit (Thermal Scientific, USA) according to its protocol, quantified by the BCA assay.

The standard detailed experimental process of the western blot was the same as our previous study (PMID29331423 and PMID29800682).

### 2.4 Statistical analysis

All data analyses were performed in R4.2.0. Pearson’s test was used to calculate the correlation between different genes. Wilcox rank sum test and Kruskal Wallis rank sum test were used for assessing differences for continuous variables. Univariate Cox regression was performed to calculate the hazard ratio (HR) and the log-rank test was used to compare survival differences. Heatmap was performed by the *pheatmap* package in R4.2.0. Receiver operating characteristic (ROC) curves and the AUC value were performed by the *pROC* package in R4.2.0. GO and KEGG analyses were performed by the *clusterProfiler* package in R4.2.0. PTPRC-correlated genes were identified in the online tool (GeneMANIA: http://genemania.org/search/homo-sapiens/mtdh). *P<0.05* was considered to indicate a statistically significant difference.

## 3 Results

### 3.1 ICD subgroups division in pan-cancer

**Figure 2:**
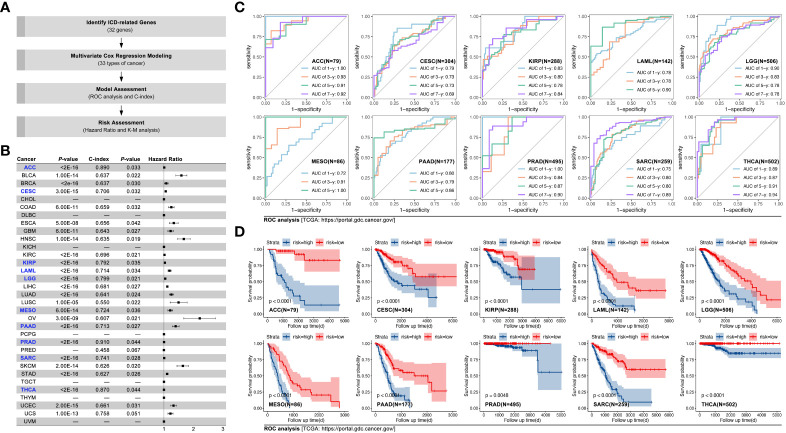
IRGs-based prognosis training model in pan-cancer. **(A)** Technology roadmap of study; **(B)** Prognosis prediction efficiency in pan-cancer, and C-index of models in ACC, CESC, KIRP, LAML, LGG, MESO, PAAD, PRAD, SARC, and THCA were greater than 0.7 (labelled by blue); **(C)** ROC curves of ACC, CESC, KIRP, LAML, LGG, MESO, PAAD, PRAD, SARC, and THCA; **(D)** Kaplan-Meier survival curves of ACC, CESC, KIRP, LAML, LGG, MESO, PAAD, PRAD, SARC, and THCA.

**Figure 3:**
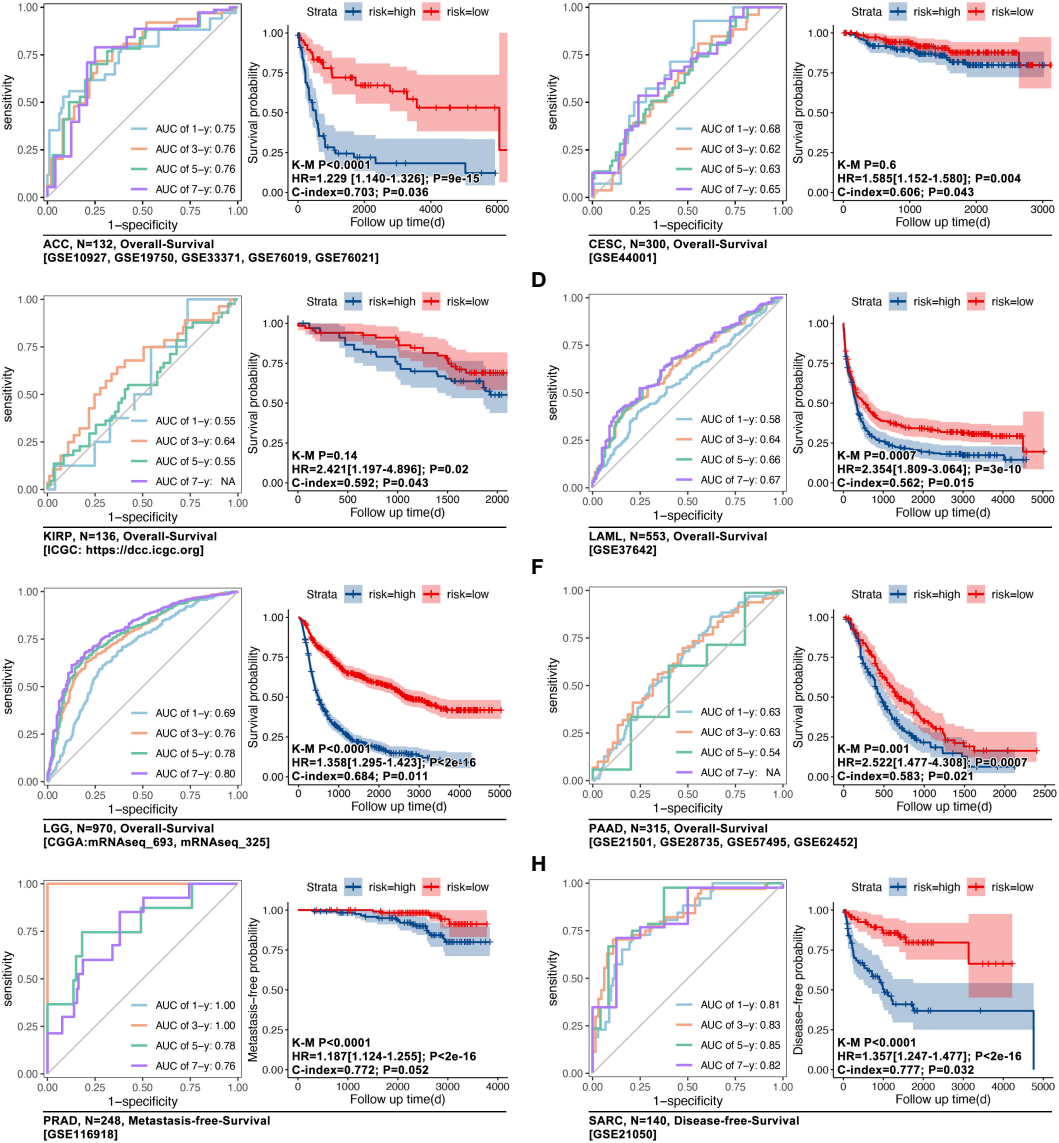
IRGs-based prognosis verifying models in eight types of cancer. GEO cohorts were used to verify TCGA-based IRGs-based prognosis models in **(A)** ACC (n=132, GSE10927, GSE19750, GSE33371, GSE76019, GSE76021), in **(B)** CESC (n=300, GSE44001), in **(C)** KIRP (n=136, ICGC), in **(D)** LAML (n=553, GSE37642), in **(E)** LGG (n=970, mRNAseq_693, mRNAseq_325), in **(F)** PAAD (n=315, GSE21501, GSE28735, GSE57495, GSE62452), in **(G)** PRAD (n=248, GSE116918), and in **(H)** SARC (n=140, GSE215050).

Amongst the 31 types of cancer, multivariate Cox regression was used to construct prognosis prediction models, based on ICD-related genes (IRGs) in the TCGA cohort. Results showed that in ACC, CESC, KIRP, LAML, LGG, MESO, PRAD, SARC, and THCA, the c-index of the model was greater than 0.700 (**Figures 2A, B**, p<0.05). The AUC value was 1.00, 0.93, 0.91, and 0.92 for overall survival time at 1, 3, 5, and 7 years in ACC (**Figure 2C**). Kaplan-Meier (K-M) analysis displayed that higher ICD-gene-based multi-genes riskscore predicted worse prognosis in above mentioned eight types of cancer (**Figure 2D**, p<0.01). Subsequently, GEO data cohorts were used for verification. For example, in ACC, the AUC value of the IRGs-based multi-genes model at overall survival time of 1, 3, 5, and 7 was 0.75, 0.76, 0.76, and 0.76, respectively (K-M p<0.0001; HR=1.229[1.140-1.326], log-rank p=9e-15; c-index=0.703, p=0.036; **Figure 3A**). The last independent verifications of GEO data were displayed in **Figure 3**.

### 3.2 IRGs-based immune subgroup division and features identification

**Figure 4:**
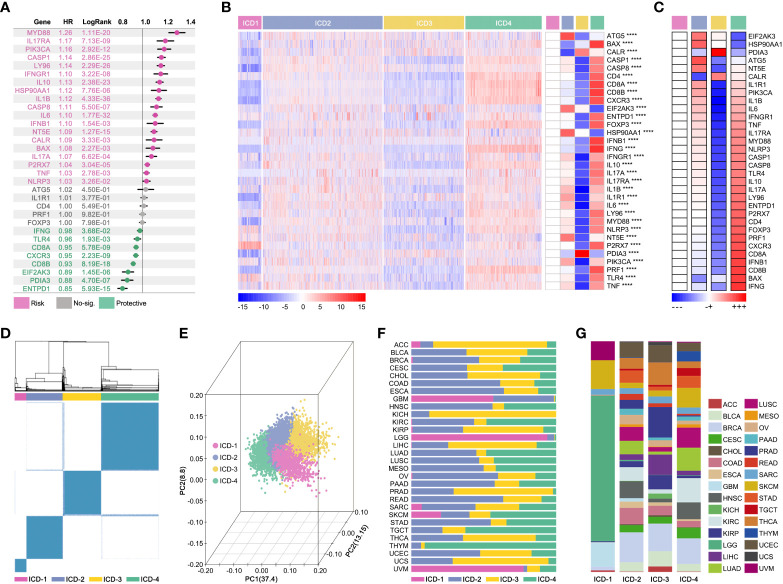
Consensus-clustering-based ICD subgroup division. **(A)** Prognosis hazard ratio (HR) of ICD-related gene in pan-cancer, calculated by univariate cox regression in R (data from TCGA). **(B, C)** Expression feature map of ICD-related genes amongst ICD subgroups, and the expression differences of ICD-related genes. **(D)** Visualization of consensus clustering. **(E)** PCoA analysis of ICG subgroups; **(F)** Proportion characteristics of ICD subgroups in pan-cancer. **(G)** Proportion characteristics of pan-cancer in ICD subgroups. ****p value < 0.0001.

The impact of IRGs on prognosis in pan-cancer was explored. As the **Figure 4A** showed, MYD88, IL17RA, PIK3CA, CASP1, LY96, IFNGR1, IL10, HSP90AA1, IL1B, CASP8, IL6, IFNB1, NT5E, CALR, BAX, IL17A, P2RX7, TNF and NLRP3 were risk factors, while IFNG, TLR4, CD8A, CXCR3, CD8B, EIF2AK3, PDIA3 and ENTPD1 were protective factors. Next, IRGs-based consensus clustering was performed to divide pan-cancer into 4 subgroups (ICD1: 753 samples, ICD2: 3835 samples, ICD3: 2565 samples, ICD4: 2440 samples). The Gene expression profile map showed expression features of IRGs amongst four subgroups, and it represented the biological characteristics of hierarchical stages of ICD (**Figures 4B, C**). In fact, visualization of consensus clustering and PCoA displayed the IRGs-based subgroup division (**Figures 1D, E**). Although the ICD subgroup spanned pan-cancer, its proportion in each cancer species was significantly different (**Figure 4F**). At the same time, the proportion of pan-cancer in different ICD subgroups was also significantly different (**Figure 4G**).

**Figure 5:**
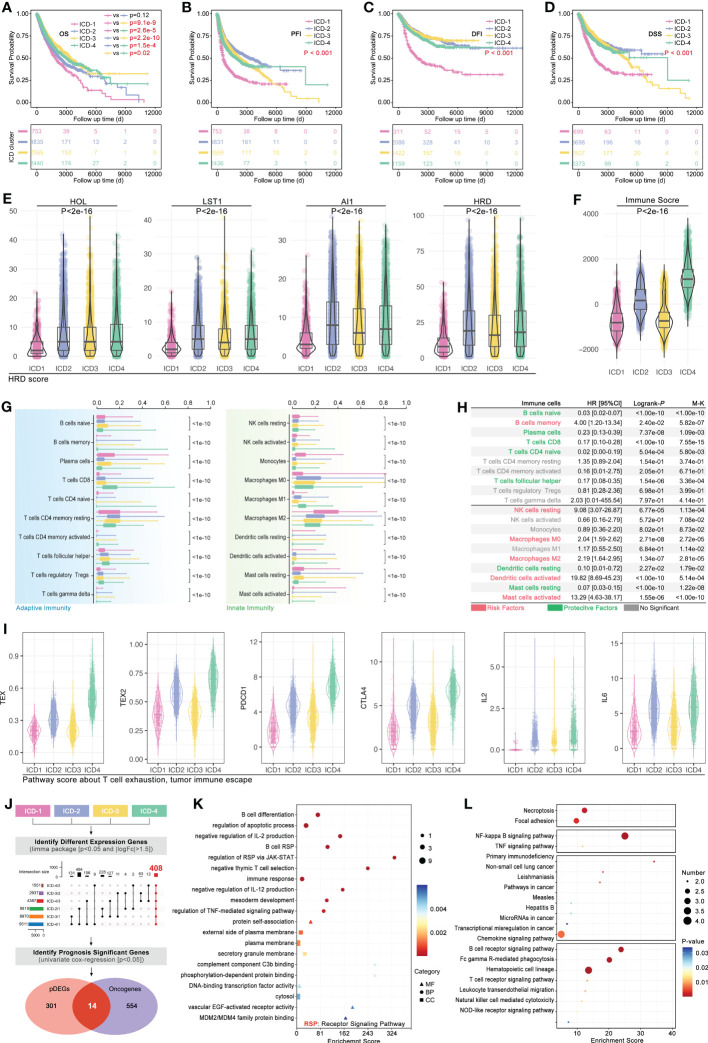
Phenotype features of ICD subgroups. Prognosis differences, including **(A)** overall survival (OS, p<0.001), **(B)** progression free interval (PFI, p<0.001), **(C)** disease free interval (DFI, p<0.001) and **(D)** disease free survival (DSS, p<0.001), amongst four ICD subgroups were calculated by 4-way log-rank. **(E)** Genomic instability was assessed by the level of **(E)** HOL, LST1, AI1, and HRD; **(F)** Immune score in ICD subgroups; **(G)** Immune cell infiltration (ICI) was predicted in CIBERSORT, and all of 20 types of immune cells in tumor filtration held significant differences amongst ICD subgroups. **(H)** Prognosis hazard ratio (HR) of immune cell infiltration in pan-cancer, which calculated by univariate cox regression in R. **(I)** T-cell exhaustion (TEX) score was calculated by ssGSEA based on GEPIA (TEX2) or literature (TEX) supplied TEX-related markers. **(J)** Firstly, different expression genes between ICD subgroups (p<0.05 and |logFc|>1.5) were identified, followed by *upset* function analysis to identify 408 genes which held different expression between any two ICD subgroups. Then, univariate cox regression was performed to identify prognosis-related genes. Next, 14 genes were identified as ICD-related cancer driver genes (ICD-TDGs). **(K)** GO and **(L)** KEGG analysis.

In order to explore the effects of the ICD subgroup on clinical features, prognosis differences, immune cell infiltration (ICI), and molecular signaling pathway were described. Pan-cancer survival analysis displayed that pan-cancer of different ICD subgroups has different overall survival (OS) time (4-way long-rank p<0.0001, **Figure 5A**), while no significant difference in OS was seen between ICD1 and ICD2 only in intro-subgroups analysis (**Figure 5A**). Besides, ICD subgroups displayed obvious differences in the progression-free interval (PFI) (p<0.001, **Figure 5B**), disease-free interval (DFI) (p<0.001, **Figure 5C**), and in disease-free survival (DSS) (p<0.001, **Figure 5D**). Collectively, all survival analyses showed the same result that samples in ICD1 had the worst prognosis.

Then, tumor immunity was explored in four ICD subgroups. As **Figure 5E** showed, genomic instability (microsatellite instability) was explored, and the results showed that HOL, LST1, AI1, and HRD were kept at different levels in ICD subgroups (p<2e-16, **Figure 5E**). Immune score showed a similar tendency with genomic instability in ICD subgroups, in which the immune score’ ranking was ICD4>ICD2>ICD3>ICD1 (p<2e-16, **Figure 5F**). Furthermore, the CIBERSORT analysis was used to explore the ICI amongst ICD subgroups. 20 types of immune cells showed different disturbances in different ICD subgroups (**Figure 5G**), such as CD8+ T cells in the ICD subgroups showed a gradual upward trend, while Macrophage M2 (M2) cells in the ICD subgroup showed a gradual downward trend. Further, the ICI-related prognosis analysis was performed, and it revealed that “B cells memory, NK cells resting, macrophages M0, macrophages M2, Dendritic cells activated and Mast cells activated” were risk factors in pan-cancer, while “B cells naïve, plasma cells, T cells CD8, T cell CD4 naïve, T cells follicular helper, dendritic cells resting and mast cell resting” were protective factors in pan-cancer (**Figure 5H**). In order to explore the correlation between ICD stratification and T-cell immunity, T-cell exhaustion (TEX) score and TEX-related factors expression were explored. As **Figure 5I** showed, the TEX score varied from ICD subtypes likewise the expression of IL2, IL6, PD-L1, and CTLA4 (**Figure 5I**).

Furthermore, from phenotype into molecular pathways, GO and KEGG analysis was performed to reveal the features of the molecular signaling pathways in ICD subgroups. Firstly, different expression genes between ICD subgroups (p<0.05 and |logFc|>1.5) were identified, which was followed by *upset* function analysis to identify 408 genes that held different expressions between any two ICD subgroups (**Figure 5I**). Then, univariate cox regression was performed to identify prognosis-related genes amongst those above 408 genes. Next, 14 genes were identified as ICD-related cancer driver genes (ICD-TDGs) (**Figure 5I**). Through GO analysis, we found those 14 genes were correlated to immunity (such as *B cell differentiation, negative regulation of IL-2/12 production, immune response, and regulation of RSP via JAK/STAT*), apoptosis, and membrane receptor signaling pathways (such as *complement component C3b binding, external side of plasma membrane*) (**Figure 5J**). Besides, KEGG analysis displayed that ICD-TDGs were related to immunity (such as *chemokine signaling pathway, B/T cell receptor signaling pathway, NK-cell-mediated cytotoxicity*), tumor cell death (*necroptosis, TNF signaling pathway*), tumor invasion (*focal adhesion*) and other pathways in cancer (**Figures 5K, L**).

### 3.3 ICD-TDGs-based AI modeling

**Figure 6:**
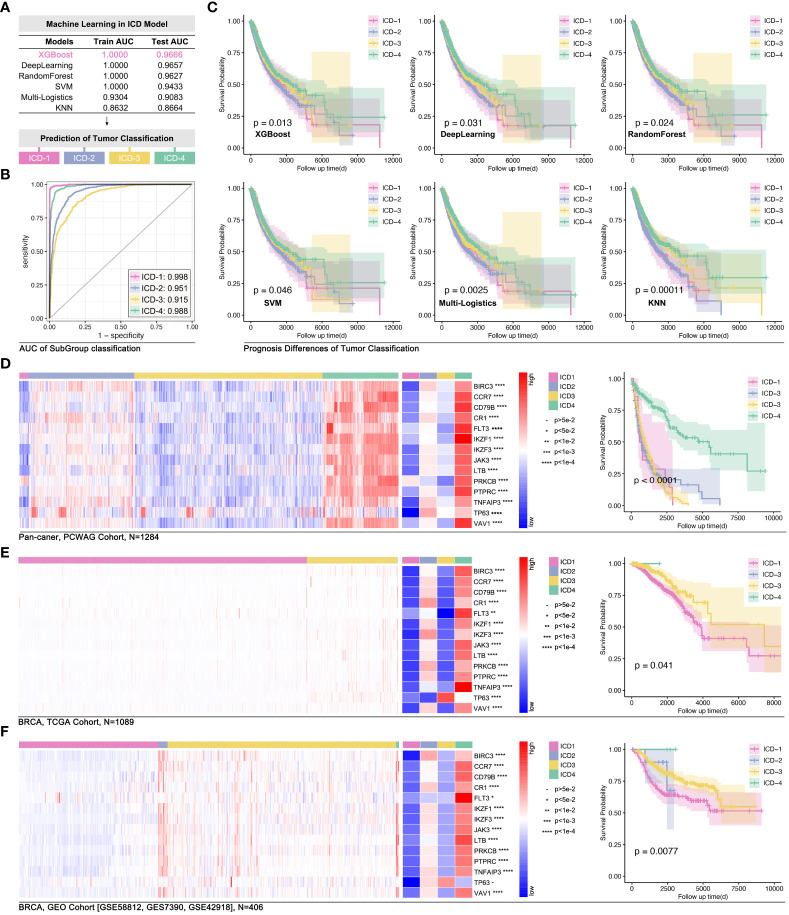
ICD-TDGs-based AI modeling identified ICD subgroups. **(A)** Randomly selecting 75% as the training cohort and 25% as testing cohort, followed by six machine learning functions (XGboost, Deep Learning, RandomForest, SVM, multi-logistics and KNN) to modeling ICD subgroups, amongst which **(B)** XGboost held best performance (training AUC=1.000, testing AUC=0.9666); **(C)** Prognosis differences between ICD subgroups modeled by XGboost (p=0.013), Deep Learning (p=0.031), RandomForest (p=0.024), SVM (p=0.046), multi-logistics (p=0.0025) and by KNN (P=0.00011). **(D)** ICD stratification in PCWAG data based on TCGA-based XGBoost model. **(E)** Gene expression feature and prognosis difference of breast cohort based on TCGA data. **(F)** Gene expression feature of ICD-TDGs and prognosis difference of ICD subgroups in GEO breast cancer cohort (n=408, GSE58812, GSE7390, GSE42918).

In order to explore the impact of 14 ICD-TDGs in the identification of ICD subgroups, artificial intelligence (AI) was used in modeling. Firstly, 75% of the pan-cancer data was randomly selected as the training cohort, and the left 25% was randomly selected as the testing cohort. In the following step, different machine learning functions were performed to identify the best one. As **Figure 6A** showed, ICD-TDGs-based XGboost showed the best modeling performance, for its training AUC was 1.000, and for testing AUC was 0.9666 (single ICD subgroup prediction AUC was 0.998, 0.951, 0.915, and 0.988 for ICD1-4, respectively, **Figure 6B**), while the values in Deep Learning (DL) was 1.000 and 0.9657, in Random Forest (RD) was 1.000 and 0.9627, in support vector machine (SVM) was 1.000 and 0.9433, in multi-logistics was 0.9304 and 0.9083, and in K-Nearest Neighbor (KNN) was 0.8632 and 0.8664 (**Figure 6A**). After that, univariate cox regression analysis was performed to assess the effects of ICD-TDGs-based AI modeling. The results showed that all six AI function-mediated modeling held significance in identifying prognosis differences in the training cohort (**Figure 6C**). Amongst the above six AI-mediated models, XGboost was finally selected for further analysis, and the ICD subgroups identification quality was 0.998 for ICD-1, 0.951 for ICD-2, 0.915 for ICD-3, and ICD-4 for 0.988 (**Figure 6B**). Then, independent cohorts were used for model verification. As **Figure 6D** showed, 14 ICD-TDGs displayed obvious expression characteristics in the pan-cancer cohort (data from PWWAG), with ICD1 with the lowest expression feature of 14 ICD-TDGs, while ICD4 with the highest expression feature (**Figure 6D**). In addition, pan-cancer model was explored in breast cancer. In order to avoid different independent groups of data bias caused by different RNA sequencing methods and batches, we transformed the gene expression value into the range of 0 to 1 (more details were shown in the method), and we defined this way as standardized ICD-TDGs-based AI model (s-AI model). As **Figure 6E** showed, the ICD1 group had the worst prognosis while the ICD4 group held the best prognosis in breast cancer (data from TCGA, p=0.041, **Figure 6E**). GEO data was used for independent verification in the breast cancer cohort. Collectively, the same results were observed (p=0.0077, **Figure 6F**).

### 3.4 ICD-TDGs-based s-AI model predicted drug sensitivity

**Figure 7:**
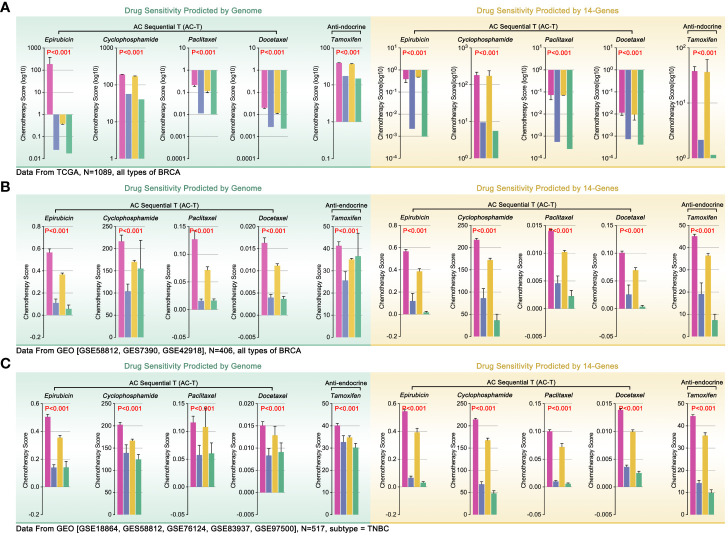
Genome-based and ICD-TDGs-based drug sensitivity prediction. **(A)** Drug sensitivity prediction, including epirubicin (EPI), cyclophosphamide (CTX), paclitaxel (PTX), docetaxel (DTX) and tamoxifen (TAM), in BRCA with TCGA data, amongst which green background represented genome-based drug sensitivity prediction while yellow background represented ICD-TDGs-based drug sensitivity prediction. **(B)** Drug sensitivity prediction in whole BRCA cohorts, in which data from GEO (GSE51561, GES20685, GSE20711, GSE25066, GSE29431, GSE613041); **(C)** Drug sensitivity prediction in TNBC cohorts, in which data from GEO (GSE18864, GES58812, GSE76124, GSE83937, GSE975001).

Differences in ICI in tumor environment were involved in tumor immune therapy. Recently, some studies linked ICI to drug tolerance, such as macrophage-mediated drug tolerance (DT) in pancreatic cancer (25, 26). So, the potential correlation between the ICD subgroups and DT was worth exploration. Firstly, TCGA genome data was used to predict drug sensitivity in BRCA by *OncoPredict* in R4.2.0. The results showed that the ICD1 and ICD3 group manifested higher drug scores (epirubicin, EPI; cyclophosphamide, CTX; paclitaxel, PTX; Docetaxel, DTX; tamoxifen, TAM), while the ICD2 and ICD4 possessed lower drug scores (higher drug score means worse drug sensitivity, **Figure 7A**). Besides, we assessed the effects of 14 ICD-TDGs as background genes in predicting drug sensitivity. Unexpectedly, the results of 14 ICD-TDGs-based drug sensitivity predictions held the same trend as the genome-based drug sensitivity prediction. That was the ICD1 and ICD3 group obtained higher drug scores while the ICD2 and ICD4 got lower drug scores (**Figure 7A**). In order to verify the above results, we performed the same analysis in GEO cohort, included whole BRCA cohort and TNBC cohort. As **Figure 7B** indicated, in the whole BRCA cohort, the ICD1 and ICD3 group still got higher drug scores while the ICD2 and ICD4 ended with lower drug scores, in the above two gene backgrounds drug prediction (**Figure 7B**). As **Figure 7C** showed, in TNBC cohort, ICD1 and ICD3 subgroups still held higher drug scores while ICD2 and ICD4 with lower drug scores, in the above two gene backgrounds drug prediction (**Figure 4C**). This unexpected phenotype implied that there was a probability of using ICD-TDGs replaced genome to predict drug sensitivity in clinical assessment.

**Figure 8:**
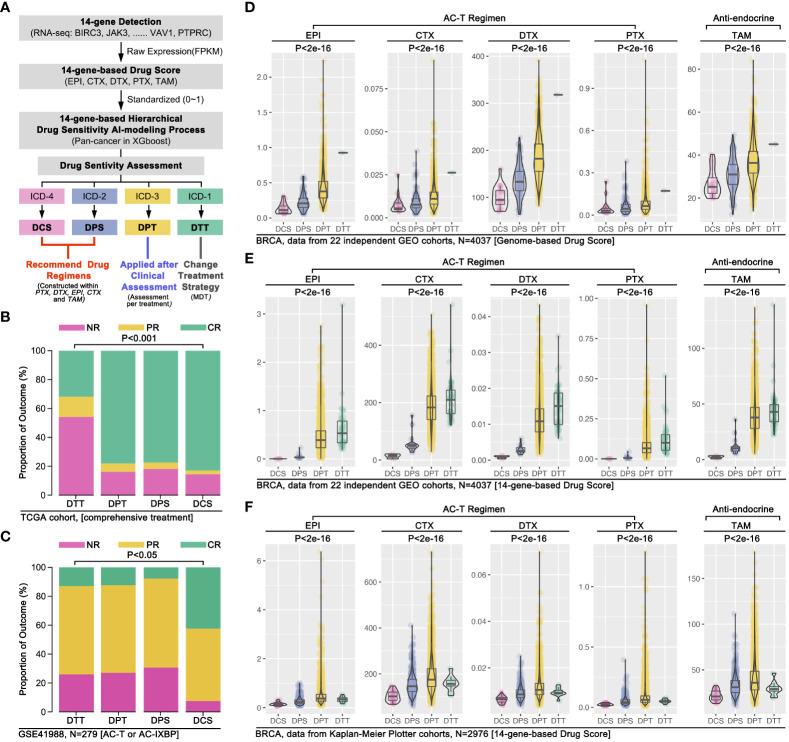
AI modeling identified drug sensitivity stratification in breast cancer. **(A)** AI modeling identified ICD subgroups were renamed as drug complete sensitivity (DCS, also named ICD4), drug partial sensitivity (DPS, also named ICD2), drug partial tolerance (DPT, also named ICD3), drug complete tolerance (DTT, also named ICD1). **(B)** Comprehensive therapy outcome in ICD subgroups, and NR mean no response, PR mean partial response, CR mean complete response; **(C)** Clinical trial therapy effects in ICD subgroups (data from GSE41988, n=279), and NR mean no response, PR mean partial response, CR mean complete response; **(D)** 22 independent breast cancer cohort from GEO database were merged into a 4037 samples cohort, followed by 14 ICD-TDGs-based AI modeling and genome-based drug prediction; **(E)** 14 ICD-TDGs-based AI modeling and 14-ICD-TDGs-based drug prediction (n=4037, GEO); **(F)** Breast cancer cohort from Kaplan-Meier Plottor was performed 14 ICD-TDGs-based AI modeling and drug prediction (n=2976).

To further verify our conjecture, we used clinical drug trials to explore the relationship between ICD subgroup division and drug sensitivity in breast cancer. Firstly, combing ICD-TDGs expression features and the results of drug sensitivity prediction, we re-defined the ICD subgroup naming, that was defining ICD4 as drug complete sensitivity (DCS) subgroup, ICD2 as drug partial sensitivity (DPS) subgroup, ICD3 as drug partial tolerance (DPT) subgroup, and ICD1 as drug complete tolerance (DTT) subgroup. The comprehensive treatment outcome was first used to assess the above AI model, and the analysis results showed that the DCS group with the best outcome while the DTT group with the worst outcome (p<0.001, **Figure 8B**). Same results were observed in GEO data (p<0.05, **Figure 8C**). Furthermore, 22 independent breast cohorts (N=4037) from GEO database were used to verify the s-AI model. Excitingly, genome-based drug sensitivity prediction showed hierarchical drug sensitivity in identified ICD subgroups, that was drug score (EPI, CTX, DTX, PTX, TAM) ranking was DCS<DPS<DPT<DTT (p<2e-16, **Figure 8D**). Meantime, 14 ICD-TDGs-based same drug sensitivity trend in identified ICD subgroups in the same cohort, that was drug score (EPI, CTX, DTX, PTX, TAM) ranking was DCS<DPS<DPT<DTT (p<2e-16, **Figure 8E**). Following, we verified the above phenotype in Kaplan-Meier Plotter breast cancer cohort, in which it displayed the drug score ranking was DCS<DPS<DPT/DTT (p<2e-16, **Figure 8E**).

In order to further verify the s-AI model, we used a non-standardized 14 ICD-TDGs expression value to construct the AI model (defined as an ns-AI model) and to assess the ability about identifying drug sensitivity stratification in ICD subgroups. The results showed that the ns-AI model identified three ICD subgroups (n=488; DCS=38, DPS=286, DPT=164, DTT=0; **Figure S1A**) and the trend of drug sensitivity was collective with the above s- AI model (**Figure S1A**). Besides, combing with clinical trial results, the ns-AI model identified hierarchical pathological complete remission (pCR) ratio in ICD subgroups (DCS>DPS>DPT, p=0.000384, **Figure S1B**). In another breast cohort (with different chemotherapy regimens), the ns-AI model identified four ICD subgroups with hierarchical drug sensitivity (n=1578; DCS=131, DPS=105, DPT=282, DTT=1060; **Figure S1C**), and the trend of drug sensitivity was collective with the above s- AI model (**Figure S1C**). Collectively, the ns-AI model identified hierarchical pCR ratio in ICD subgroups (DCS>DPS>DPT>DTT, p=0.006, **Figure S1D**).

### 3.5 Impact of immune cell infiltration in regulating drug sensitivity in breast cancer

**Figure 9:**
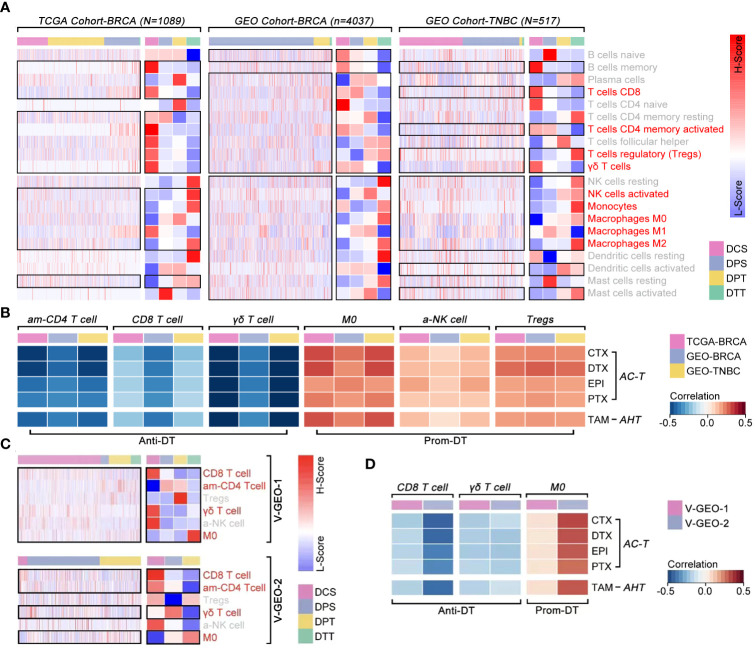
The correlation between immune cell infiltration and drug sensitivity. **(A)** Differences of ICI amongst ICD subgroups, in which *T cell CD8*, *T cell CD4 memory activity*, *Tregs, γδ T cell*, *NK cell activated*, *monocytes*, *macrophages M0*, *macrophages M1* and *macrophages M2* held different infiltration proportion in BRCA (p<0.05) (data from TCGA, GEO cohort-4037 and GEO cohort-TNBC). **(B)** *Pearson* test identified *T cell CD4 memory activity (am-CD4 T cell)*, *T cell CD8 (CD8 T cell)* and *γδ T cell* were negative correlated with drug score (p<0.05), while *macrophages M0 (M0)*, *NK cell activated (a-NK cell)* and *Tregs* were positive correlated with drug score (p<0.05) (data from TCGA, GEO cohort-ALL and GEO cohort-TNBC). **(C)** Differences of ICI amongst ICD subgroups, in which *CD8 T cell*, *am-CD4 T cell*, *γδ T cell* and *M0* held different infiltration proportion in BRCA (p<0.05) (data from GEO cohort-1 and cohort-2). **(D)** *Pearson* test identified *CD8 T cell* and *γδ T cell* were negative correlated with drug score (p<0.05), while *M0* was positive correlated with drug score (p<0.05) (data from GEO cohort-1 [n=488] and GEO cohort-2 [n=1578]).

Although previous evidence revealed that ICI was correlated with drug tolerance, there was still without convincing evidence to support its phenotype in breast cancer. Combining with the TCGA cohort and GEO cohort, we found “*T cell CD8 (CD8 T cell), T cells CD4 memory activity (am-CD4 T cell), Tregs, γδ T cells, NK cells activity (a-NK cell), monocytes, M0, M1, and M2*” held different infiltration proportion in different drug sensitivity subgroups (p<0.05, **Figure 9A**). Following, Pearson-correlated test was performed to identify that “*am-CD4 T cell, CD8 T cell, and γδ T cell*” were negatively correlated with drug score, which means higher infiltration of those cell enhanced drug sensitivity (p<0.001, **Figure 9B**). On the contrast, “*M0, a-NK cell and Tregs*” were positively correlated with drug score, which means those cell infiltrations enhanced drug tolerance (p<0.001, **Figure 9B**). Then, we verified the relationship between those above six immune cells with drug score. As the results showed, “*CD8 T cell, am-CD4 T cell, γδ T cell, and M0”* were different expressions in breast cancer, amongst which “*CD8 T cell, γδ T cells, and M0”* were correlated with drug score (**Figures 9C, D**).

### 3.6 PTPRC correlative with CD8+ T cell infiltration

**Figure 10:**
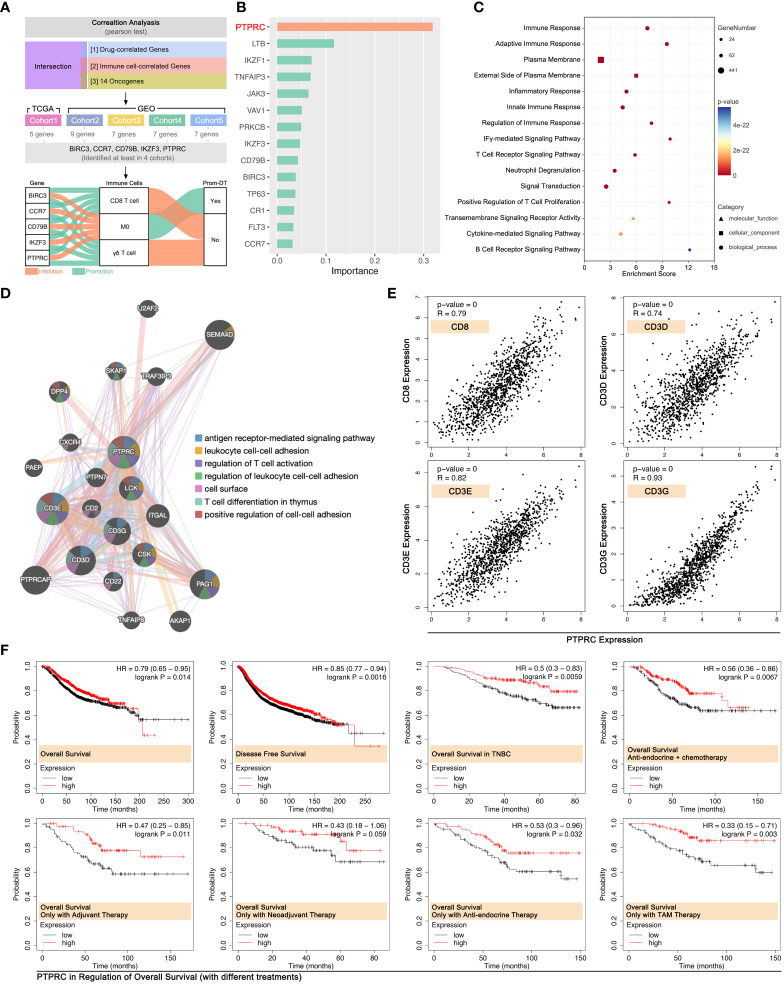
PTPRC was pivotal in CD8 T cell infiltration in BRCA. **(A)** Identifying both drug score related and immune cell infiltration related ICD-TDGs in four independent cohort (TCGA-BRCA, GEO cohort-4037, GEO cohort-TNBC, GEO cohort-1, GEO cohort-2), then identifying BIRC3, CCR7, CD79B, IKZF3 and PTPRC were the common feature genes in four data cohorts. All of those five genes were positively corelated to *CD8 T cell* and *γδ T cell*, while they were negatively corelated to *M0*. **(B)** Impact analysis in XGBoost Modeling; **(C)** GO analysis of PTPRC-related genes; **(D)** Molecular pathways of PTPRC-related genes (GeneMANIA: http://genemania.org); **(E)** Linear curve between PTPRC and markers of CD8+ T cells (CD8, CD3D, CD3E, CD3G); **(F)** Roles of the PTPRC expression level in prognosis of breast cancer with or without drug treatments.

To further cleared the pivotal genes in the regulation of immune cell infiltration-mediated drug tolerance, multiple Pearson’s tests were performed in multiple independent data cohort. As **Figure 10A** showed, BIRC3, CCR7, CD79B, IKZF3, and PTPRC were finally identified from 14 ICD-TDGs, which were positively correlated with *γδ T cell* and *CD8 T cell* infiltration while they were negatively correlated with *M0* infiltration (**Figure 10A**). Meanwhile, those five genes were negatively correlated with drug score. Following, impact ranking analysis was performed to identify the key gene in AI modeling. As **Figure 10B** implied, PTPRC was the most important gene, and it was selected for further analysis. Following, PTPRC-associated genes were explored, including interaction, co-expression, and co-localization. Those genes were related to immune response, T cell receptor signaling pathway, cytokine-mediated signaling pathway, etc. (**Figure 10C**). Besides, PTPRC was predicted to interact with CD3D, CD3E, and CD3G, and those were markers of *CT8 T cells* (**Figure 10D**). In order to explore the relationship between PTPRC and *CD8 T cell* infiltration, the Pearson test was performed, and the results showed that PTPRC was closely correlated with the expression level of CD8 (R=0.79, p<2e-16), CD3D (R=0.74, p<2e-16), CD3E (R=0.82, p<2e-16) and CD3G (R=0.93, p<2e-16) (**Figure 10E**).

Effects of PTPRC in the regulation of prognosis were explored. The results showed that a higher expression level of PTPRC was accompanied by the better outcome in OS and DFS in the whole BRCA cohort (data from Kaplan Meier-plottR), and the same result was observed in the TNBC cohort (data from Kaplan Meier-plottR) (p<0.05, **Figure 10F**). Furthermore, the effects of PTPRC in the regulation of prognosis in different drug regimens cohorts were revealed, and the results showed that higher expression level of PTPRC was accompanied with better prognosis, no matter in cohort with single chemotherapy strategy or in cohort with comprehensive therapy regimens (anti-endocrine and chemotherapy) (**Figure 10F**).

### 3.7 PTPRC promoted CD8+ T cell infiltration and enhanced PTX-induced tumor death

**Figure 11:**
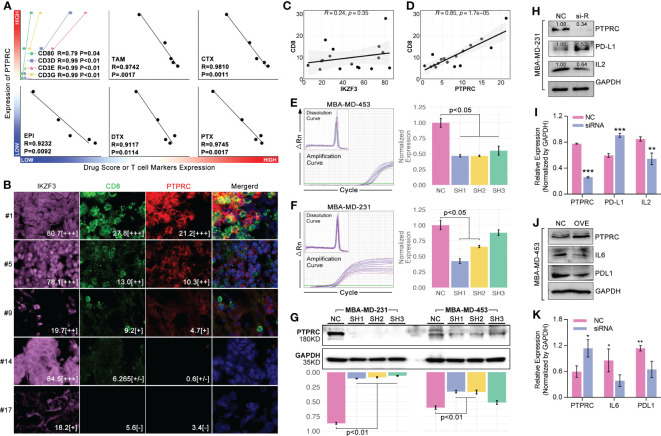
PTPRC regulated CD8 T cell infiltration and TEX. **(A)** Local TNBC cohort was performed 14-ICD-TDGs-based drug sensitivity prediction, and the relationships between PTPRC and markers of CD8+ T cells, and between PTPRC and drug scores were explored; **(B)** Multiple immune inflorescence staining was performed in breast cancer tissues, amongst which pink represented IKZF3, green represented CD8, red represented PTPRC. And the results displayed the quantity of **(C)** IKZF3+ cells were not related with CD8+ T cells, while **(D)** PTPRC+ cells were positively related with CD8+ T cells (R=0.85, p=1.7e-5); TR-qPCR assays of siRNA experiments in TNBC cell lines, included **(E)** MBA-MD-453 and **(F)** MBA-MD-231; **(G)** Western blot assays displayed siRNA explements results, and sequence 1(SH1) and sequence-2 (SH2) were both efficient in decreased the intracellular expression level of PTPRC in TNBC cell lines; **(H, I)** siRNA and **(J, K)** recombination plasmid were applied to decrease the expression of PTPRC to explore the roles of PTPRC in regulating IL2/6 and PDL1. *p < 0.05; **p < 0.01; ***p < 0.001.

In order to further verify the ability of 14 ICD-TDGs-based s-AI models in predicting drug sensitivity, and to verify the impact of CD8+ T cells in regulating drug sensitivity in breast cancer, *in vitro* experiments were performed. Firstly, we collected breast samples to perform an RNA sequence assay to gain a genome expression profile, which was further used to calculate drug score and obtain the expression level of CD8+ T cell markers. As the results displayed, markers of CD8+ T cells were closely correlated with the expression of PTPRC (p<0.05, **Figure 11A**), and drug score (TAM, CTX, EPI, DTX, and PTX) were negatively related with the expression of PTPRC (p<0.05, **Figure 11A**). Then, multiple immune fluorescence staining (MIF) of breast cancer tissue sections showed that the infiltration ratio of CD8+ T cells was positively closely related with the expression level of PTPRC (R=0.85, P=1.7e-05, **Figures 11C, D**), while there was no obvious relationship between CD8+ T cells and IKZF3 (R=0.24, P=0.35, **Figures 11C, D**). To further explore the effects of PTPRC in regulating T cell immunity, we applied siRNA system and recombination plasmid to decrease or increase the expression of PTPRC, respectively. As the results showed, the down-regulation of PTPRC increased the tumoral (MBA-MD-231) expression of PD-L1 about 1.53-fold as compared to NC group, while the expression level of tumor-derived IL2 was down-regulated (**Figures 11H, I**). Meantime, the tumor-derived IL6 and PD-L1 were down-regulated as the up-regulation of PTPRC (**Figures 11J, K**).

**Figure 12:**
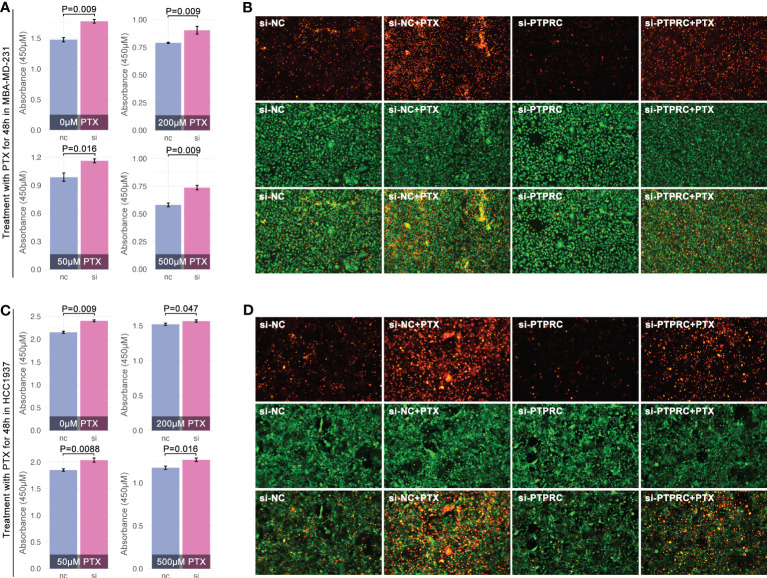
PTPRC regulated PTX sensitivity in TNBC. Decreasing of PTPRC by siRNA was followed by PTX treatment in MBA-MD-231 **(A)** and MBA-MD-453 **(C)**, and viability was detected by CCK8. **(B, D)** Alive&death assay was performed to detect proportion of alive or dead cells with different treatments (orange means dead cells, while green means alive cells).

Next, we explored PTPRC in regulating PTX sensitivity. As results showed, intracellular expression of PTPRC was obviously decreased by siRNA sequence1 (SH1) in TNBC cell lines (MBA-MD-231, MBA-MD-453) (**Figures 11E–G**). Following, cell cytotoxicity assays were performed, and the results displayed that the down-regulation of intracellular expression of PTPRC was accompanied by up-regulation of cell viability (p=0.009, PTX=0μM, **Figures 12A, C**). Meantime, down-regulation of intracellular expression of PTPRC led to PTX tolerance in TNBC in a dose of 50μM, 200μM, and 500μM (p<0.05, **Figures 12A, C**). Alive&death assay was applied to detect the proportion of alive cells and dead cells after siRNA treatment, PTX treatment, or the combination treatment in TNBC cell lines. Collectively, down-regulation of PTPRC decreased the proportion of dead cells induced by PTX (**Figures 12B, D**).

### 3.8 ICD-TDGs and clinical features-based prognosis model in breast cancer

**Figure 13:**
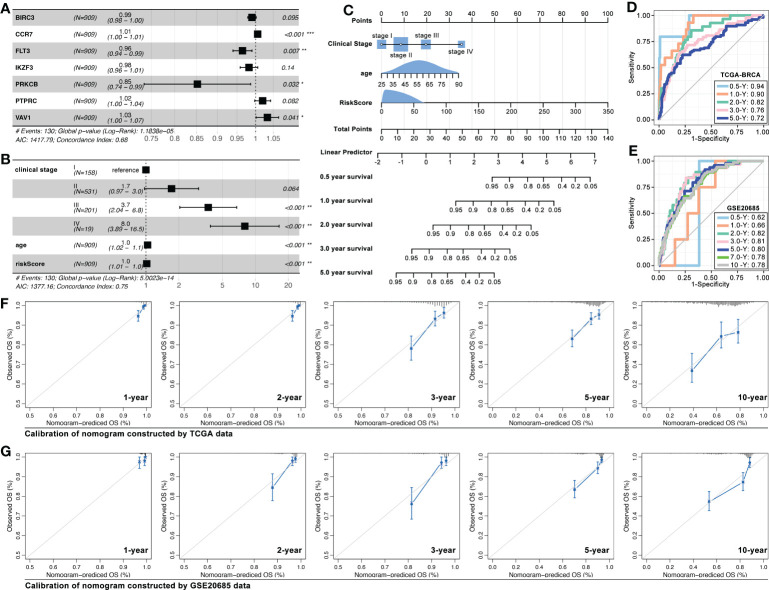
ICD-TDGs combined with clinical features to construct nomogram. **(A)** Multivariate cox regression identified BIRC3, CCR7, FTL3, IKZF3, PRKCB, PTPRC, and VAV1 to construct multi-gene riskscore; **(B, C)** Riskscore, clinical stage and age constructed nomogram; **(D, E)** AUC values of training cohort (TCGA-BRCA) and testing cohort (GEO20685); Calibrations of nomogram based on **(F)** TCGA data and **(G)** GEO data. *p < 0.05; **p < 0.01; ***p < 0.001.

Recently, ICD-associated genes were used to construct a prognosis model in melanoma, lung cancer, and ovarian cancer (27–29). So, we explored the ICD-TDGs in constructing a prognosis model with clinical features in breast cancer. Multivariate cox regression identified BIRC3, CCR7, FLT3, IKZF3, PRKCB, PTPRC, and VAV1 to construct multi-gene risk-score (MGRS) (C-index=0.68, p=1.1838e-5, **Figure 13A**). Then, clinical stage, age, and MGRS were identified to construct nomogram (C-index=0.75, p=5.0e-14, **Figures 13B, C**). And the AUC value of 6-month survival, 1-year survival, 2-year survival, 3-year survival, and 5-year survival was 0.94, 0.90, 0.82, 0.76, and 0.72, respectively (data from TCGA, **Figure 13D**). Next, GEO data was set as verification cohort, and the AUC value of 6-month survival, 1-year survival, 2-year survival, 3-year survival, 5-year survival, 7-year survival, and 10-year survival was 0.62, 0.66, 0.82, 0.81, 0.80, 0.78, and 0.78, respectively (data from GSE20685, **Figure 13E**). To further verified the prediction accuracy of the above nomogram, calibration assay was performed, and the results were displayed in **Figures 13F, G**.

## 4 Discussion

Breast cancer is the most common type of cancer among women, and also the main cause of tumor-related deaths in women (30). Drug therapy is important for cancer elimination, and also a major strategy to save advanced breast cancer. Although combing treatment of surgical operation and drug therapy, more than 30% of early breast cancer has developed into advanced breast cancer, which results in a 5-year overall survival rate of less than 20% (31). ICD is reported to be positively related to clinical drug therapy response, especially for immune therapy (7). Besides, ICD-associated genes are related to survival prognosis in multiple cancers (27–29). So it is worth exploring the ICD in constructing a prognosis prediction model and ICD-mediated drug sensitivity prediction model.

In previous studies, IRGs-based prognosis models were explored in melanoma, lung cancer, and ovarian cancer (27–29), while in our study pan-cancer, the bioinformatic analysis showed IRGs-based prognosis models held relatively better prediction ability in ACC, CESC, KIRP, LAML, LGG, MESO, PRAD, SARC, and THCA (C-index of the model was greater than 0.700, **Figures 2A, B**, p<0.05). Furthermore, models in SARC and PRAD displayed better prediction ability in a testing cohort (data from GEO, **Figures 3G, H**). Those results imply that IRDs-based prognosis models may be more useful to be applied in PRAD and SARC in clinical treatment. Further to explore the roles of ICD in identifying pan-cancer subgroups, artificial intelligence (AI) was applied in the following work.

AI was wildly applied in medical fields, such as automatic diagnosis (medical image, electrocardiograph, etc.), drug discovery, and clinical outcome prediction (32, 33). Recently, AI was applied in the identification of tumor subgroups and drug sensitivity prediction. For instance, T-cell exhaustion-related genes were used to identify immunity subgroups in pan-cancer, and it contributed to making anti-immunity therapy strategy in melanoma (34). Interestingly, in this study, we found IRGs-based AI modeling (XGBoost, deep learning, random forest, SVM, multi-logistics, and KNN) identified prognosis subgroups (**Figures 6A–C**), which was verified in the testing cohort in breast cancer (**Figures 6E, F**).

ICD was reported to regulate chemotherapy sensitivity in bladder cancer, lung cancer, and pancreatic cancer (35–37), and it was also related to anti-PD1 therapy and chemotherapy in breast cancer (38, 39). Although IRGs were already used to construct a prognosis model in breast cancer before (40–42), IRGs-based drug sensitivity stratification was not clear yet now. In our study, *OncoPredict* package was applied to predict drug sensitivity, which showed the trend of genome-based drug sensitivity prediction was as same as 14-ICD-TDGs-based drug sensitivity prediction (ICD1>ICD3>ICD2>ICD4), and this phenotype was verified in the TCGA cohort (n=1089), GEO cohort (n=406, breast cancer without subtypes), and TNBC GEO cohort (n=517) (**Figure 7**). The above drug sensitivity stratification was re-defined as DTT (ICD-1), DPT (ICD-3), DPS (ICD-2), and DCS (ICD-4), amongst which DCS and DPS subgroups were recommended to accept drug treatment, while DPT subgroup was recommended to accept advanced clinical assessment for drug treatment, and DTT subgroup was recommended to change treatment strategy (excluded AC-T regimen) (**Figure 8A**). Those drug therapy strategies were verified in clinical trials and big data bioinformatic analysis. Both of TCGA-derived clinical cohort (1089) and GEO-derived clinical trial cohort (GSE41988, N=279) showed similar drug sensitivity trend in ICD subgroups as 14-ICD-TDGs-based drug sensitivity prediction (**Figures 8B, C**). Furthermore, in 22 independent breast cancer cohorts (4037 samples), the same results were observed (14-ICD-TDGs-based drug sensitivity prediction represented genome-based drug sensitivity prediction) (**Figures 8D–F**). The above results implied that 14 ICD-TDGs were worth being applied in clinics to assess drug sensitivity to make drug therapy regimens for patients with breast cancer, which was like the role of the 21-gene test in clinical drug therapy for breast cancer.

To further uncover the mechanisms of ICD-mediated regulation of drug sensitivity, tumor immunity differences were explored. Genomic instabilities, including tumor mutation burden (TMB), genomic alteration fraction, and microsatellite instability, were related to tumor immunity (43). We found there were significant differences in microsatellite instability between ICD subgroups, and the scoring trend of them was ICD1<ICD3<ICD2/ICD4 (**Figure 5E**). Collectively, immune score ranking in ICD subgroups was ICD1<ICD3<ICD2<ICD4 (**Figure 5F**). Interestingly, the trend of immune score amongst ICD subgroups was as same as that in drug sensitivity prediction. That implied ICD-mediated regulation of tumor immunity was probably related with ICD-mediated regulation of drug sensitivity. In fact, previous studies had reported immune cell infiltration was closely related to chemotherapy. To our knowledge, CD8+ T cell infiltration was important in regulating immune therapy (anti-PD1 therapy) (44–46) and chemotherapy resistance (47, 48). In breast cancer, the maintenance of functional T-cell responses was reported as a critical requirement for neoadjuvant-chemotherapy-induced pCR (49). In our study, impact analysis showed PTPRC was the pivotal gene in AI modeling of ICD subgroup identification (**Figure 10B**), and it was positively related to the CD8+ T cell infiltration (**Figure 10A**) and markers of CD8+ T cell in breast cancer (TCGA data, **Figure 10E**; local cohort, **Figure 11A**). In addition to bioinformatic analysis, MIF assays also showed that the expression strength of PTPRC in breast cancer cells was positively related to CD8 expression strength in breast cancer tissue section (R=0.85, p=1.75e-5, **Figures 11B, D**). Meantime, the expression level of PTPRC was positively related to drug sensitivity (EPI, CTX, DTX, PTX, TAM) (TCGA data, **Figure 10A**; local cohort, **Figure 11A**), and it was also a protective factor in the clinical treatment of breast cancer patients with neoadjuvant therapy or anti-endocrine therapy (**Figure 10F**). To our knowledge, PTPRC mutation was related to the sensitivity of selective JAK inhibitor in acute lymphoblastic leukemia (50), and combined drug treatment (JQ1 and GSK2801) made synergistic proliferation inhibition by probable interaction with PTPRC in a molecular modeling study in TNBC (51). Those phenotypes implied PTPRC probably regulated drug sensitivity through CD8+ T cell infiltration in breast cancer. Excitingly, *in vitro* experiments displayed that down-regulation of intracellular expression of PTPRC exactly enhanced the PTX tolerance in TNBC cell lines (**Figures 12A–D**). Besides, down-regulation of PTPRC lead down-regulation of tumor-derived IL2 and up-regulation of tumoral PD-L1 (**Figure 11H**). Those above results implied that PTPRC played pivotal roles in regulating T-cell immunity-mediated chemotherapy sensitivity in TNBC.

## 5 Conclusion

Through bioinformatic analysis and *in vitro* experiments, some interesting and unexpected phenotypes are revealed: (1) 14 ICD-TDGs-based AI model identified hierarchical prognosis in pan-cancer, and it was also consistent with genome-based drug sensitivity stratification in breast cancer; (2) Immune cell infiltration is related with drug tolerance, amongst which *CD8+ T cell* infiltration was pivotal in regulating ICI-induced drug tolerance in breast cancer; (3) PTPRC is a protective factor in regulation *CD8+ T cell-*mediated drug sensitivity in breast cancer; (4) ICD-TDGs and clinical features constructed a nomogram which held the ability to predict prognosis in breast cancer.

However, the mechanisms of PTPRC-mediated promotion of CD8+ T cell infiltration were still not clear, and further work was needed to uncover this night veil.

## Data availability statement

The datasets presented in this study can be found in online repositories. The names of the repository/repositories and accession number(s) can be found below: GSA *via* accession ID: HRA004621.

## Ethics statement

The studies involving human participants were reviewed and approved by the Medical Ethics Committee of The First People’s Hospital of Xiaoshan District, Xiaoshan Affiliated Hospital of Wenzhou Medical University. The patients/participants provided their written informed consent to participate in this study.

## Author contributions

ZW and HJ contributed to the conception and design of this research. PL and TP performed data analysis, respectively. WS and WW performed additional experiments *in vitro*, and give professional language modification. YQH and HW performed in vitro experiments. WZ and GC performed data verification. PL and YTH made the original manuscript; ZW made the final manuscript. All authors approved the submission.

## Supplementary material

Supplementary Figure 1 Drug sensitivity stratification. **(A)** GEO cohort-1 (GSE25066, GSE25055, GSE25065) with neoadjuvant (taxane-anthracycline) was identified into DCS, DPS and DPT subgroups by ICD-TDGs-based AI modeling, amongst which DCS subgroup was most sensitive to drug therapy (p<2.22e-16), while DPT was most resistant to drug therapy (p<2.22e-16). **(B)** ICD subgroups identified obvious differences of taxane-anthracycline regimen-mediated pathological complete remission (pCR), amongst which in DCS subgroup pCR was 55.3%, in DPS subgroup pCR was 43.4%, and in DPT subgroup pCR was 27.4% (p=0.000384). **(C, D)** Similar results were observed in GEO cohort-2, in which different independent cohorts with different chemotherapy regimens.
