# Supplementary figures and images for: PTPRC promoted CD8+ T cell mediated tumor immunity and drug sensitivity in breast cancer: based on pan-cancer analysis and artificial intelligence modeling of immunogenic cell death-based drug sensitivity stratification
Source: Front Immunol. 2023 Jun 14;14:1145481. doi: 10.3389/fimmu.2023.1145481 (PMC10302730; doi:10.3389/fimmu.2023.1145481)

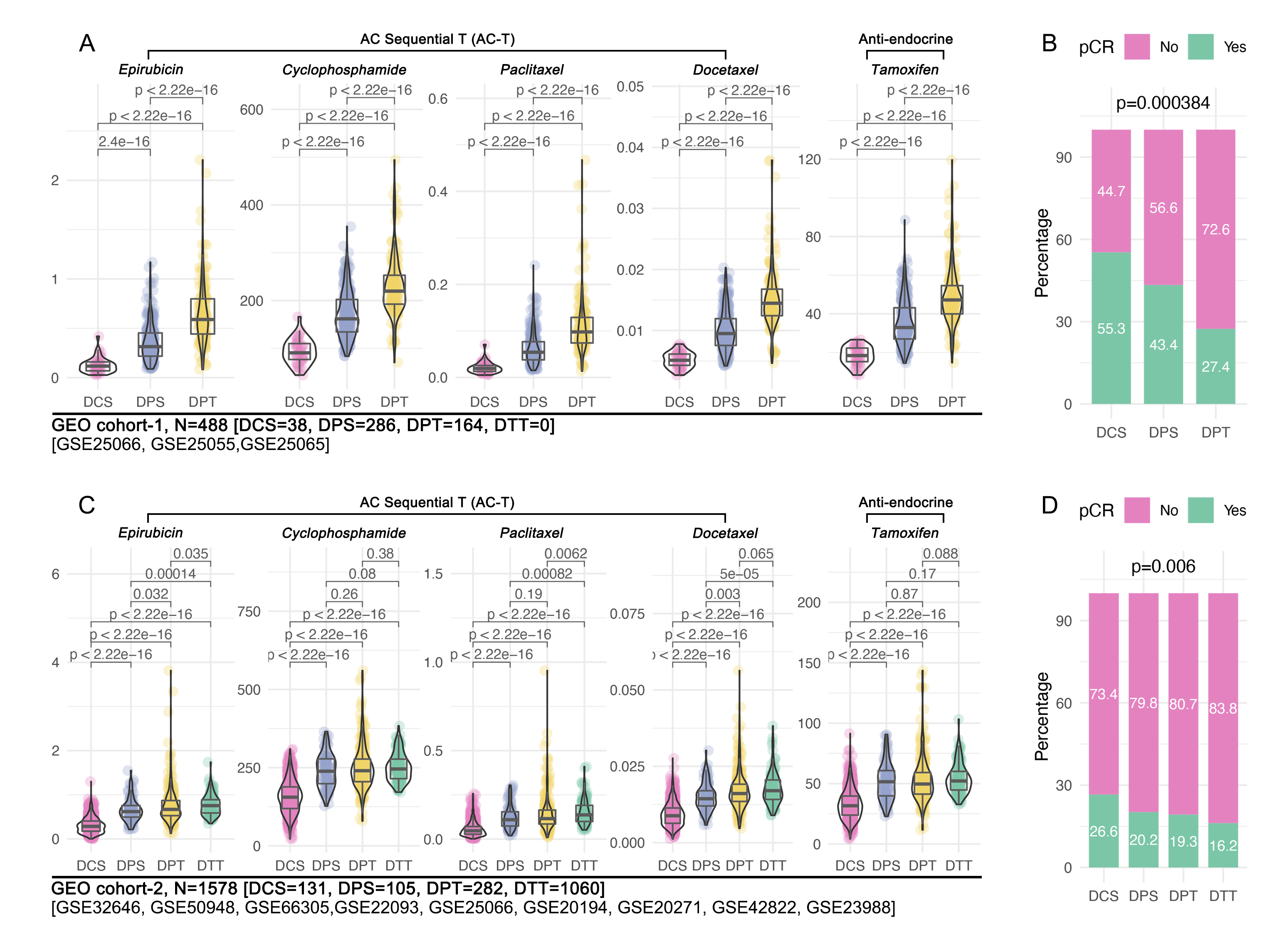

Supplement: Supplementary Figure 1 — Drug sensitivity stratification. (A) GEO cohort-1 (GSE25066, GSE25055, GSE25065) with neoadjuvant (taxane-anthracycline) was identified into DCS, DPS and DPT subgroups by ICD-TDGs-based AI modeling, amongst which DCS subgroup was most sensitive to drug therapy (p<2.22e-16), while DPT was most resistant to drug therapy (p<2.22e-16). (B) ICD subgroups identified obvious differences of taxane-anthracycline regimen-mediated pathological complete remission (pCR), amongst which in DCS subgroup pCR was 55.3%, in DPS subgroup pCR was 43.4%, and in DPT subgroup pCR was 27.4% (p=0.000384). (C, D) Similar results were observed in GEO cohort-2, in which different independent cohorts with different chemotherapy regimens. [file Image_1.tif]
